# Differences and Associations between Physical Activity Motives and Types of Physical Activity among Adolescent Boys and Girls

**DOI:** 10.1155/2022/6305204

**Published:** 2022-05-31

**Authors:** Karel Frömel, Dorota Groffik, Michal Šafář, Josef Mitáš

**Affiliations:** ^1^Institute of Active Lifestyle, Faculty of Physical Culture, Palacký University Olomouc, Tř. Míru 115, 771 11 Olomouc, Czech Republic; ^2^Institute of Sport Sciences, The Jerzy Kukuczka Academy of Physical Education in Katowice, Mikołowska 72a, 40-065 Katowice, Poland

## Abstract

Interventions aimed at motivation for physical activity (PA) are mostly beneficial, but the effects on preventing the decrease in PA are not entirely clear, especially in girls. The main aim of this study was to identify the differences and associations between PA motives and types of PA in boys and girls and between low and high motivated boys and girls. Another aim is to identify the types of motivation and PA that increase the likelihood of achieving PA recommendations and to propose ways of increasing PA among low motivated adolescents. The research carried out before the COVID-19 pandemic (2010–2019) and involved 2,149 Czech and 1,927 Polish adolescents aged 15–19 years. The International Physical Activity Questionnaire-Long Form was used to identify the level of PA types, while PA motivation was examined through the Motives for Physical Activities Measure-Revised. During the ten years, a decline was observed in enjoyment, fitness, and social motives. An increase in appearance motives was observed in girls, while no significant changes were seen in boys. Boys showed a higher motivation for PA than girls in enjoyment, competence, fitness, and social motives, while girls were high motivated in appearance motives. The greatest statistically significant differences between low and high motivated individuals were found in the associations between recreation/vigorous PA and between all types of motivation in boys and girls in both countries. The strongest associations in both genders were observed between enjoyment/competence motives and recreation/vigorous PA. Respecting and using the associations between the types of PA motives and types of PA in low and high motivated boys and girls can support feelings of PA enjoyment, increase PA, support the achievement of PA recommendations, and positively affect adolescents' healthy lifestyles.

## 1. Introduction

The increasing importance of successful physical activity (PA) motives is unequivocal for a healthy lifestyle [1], mental and physical fitness [2–4], increased participation in organized and group-based PA [5, 6], suitable climate for PA in physical education lessons [7], and children's and adolescents' well-being [8, 9]. The number of interventions that use PA motives to support children's and adolescents' PA is similarly increasing [10–12]. Interventions aimed at motivation for PA and increase in PA are mostly beneficial, but the effects are not entirely clear in terms of their effectiveness [13–15]. Various studies suggest that a high degree of heterogeneity [16] and evidence is often limited [17]. Furthermore, there is limited evidence suggesting that interventions aimed at children, adolescents, and young adults are effective when provided that PA-related enjoyment increases [11].

There is abundant evidence concerning suitable approaches to PA motives based on the self-determination theory [18] and proposals for the use of a combination of the affective-reflective theory of physical inactivity and exercise [19] and the theory of energy cost minimization [20]. These theories emphasize the need for deeper research concerning the types of PA motives and the importance of physical load optimization. They also assert feelings of PA satisfaction, immediate identification, and use of positive or negative PA assessment or physical inactivity, as well as clear identification of positive assessment in association with pleasure or negative assessment in association with displeasure [21]. Furthermore, in line with the self-determination theory, Dishman et al. [22] accentuate the need to focus on autonomous motivation while respecting external motivation and support a parallel focus on specific objectives such as appearance, competence, enjoyment, fitness, or social factors. Numerous studies also emphasize the need for further research aimed at intrinsic motivation to enhance adolescents' daily moderate to vigorous PA (MVPA) [23], identifying individuals with low intrinsic motives for PA [24] and removing gender imbalance in PA levels and autonomous forms of motivation to encourage physical activities in adolescent girls [25].

In a ten-year cross-section research study, we strive to create starting points that can support more effective motivation of girls and boys for PA in challenging postpandemic times. Due to the unsatisfactory findings in the trends of PA in adolescents [26], we focus more on girls and adolescents with less motivation for PA. Furthermore, PA motives and types of PA are characteristics of these adolescents. We consider respecting different motives and different types of PA to be essential. To increase the strength of the study, we conducted the same research in different educational environments of Czech and Polish adolescents. The research is aimed at answering the following questions:

- What changes took place during the long-term monitoring of PA motives and PA types in boys and girls?
- What are the associations of PA types and PA motives with low-and high-motivated boys and girls?
- What are the associations between PA motives, PA types, and achievement of PA recommendations?

The study should help to resolve the question for supporting the right selection of the type of motivation in boys and girls to different types of physical activity.

The main aim of this study was to identify the differences and associations between PA motives and types of PA in boys and girls and between low and high motivated boys and girls. Another aim is to identify the types of motives and PA that increase the likelihood of achieving PA recommendations and to propose ways of increasing PA among low motivated adolescents.

## 2. Materials and Methods

### 2.1. Participants and Setting

This retrospective cross-sectional study was carried out in 68 secondary schools in the Czech Republic and 76 secondary schools in Southern Poland between 2010 and 2019. The schools were selected based on long-term cooperation with the university departments. Two coeducational classes of students were randomly selected from each selected school. The entire research was performed by the same research teams in both countries, always accompanied by a responsible administrator designated by the school management. Each year, the research, involving 1,558 boys and 2,518 girls (Table 1), was carried out in five to eight schools on an average in both countries.

Body mass index (BMI) was calculated using the WHO BMI *z*-scores for adolescents [27]. Out of the total, 19.3% of boys and 11.4% of girls were observed to be overweight or obese. The numbers of participants in fall (September–November) and spring (March–May) during the study periods were similar. School management, parents, and participants provided their written consent to participate in the study. Regarding the fact that the research was presented as part of education and a source of important information for school management, the research included all students in the selected groups, who were present on the day of the research.

### 2.2. Measures

The PA motives were identified using the Motives for Physical Activity Measure-Revise (MPAM-R) scale [28]. Both the Czech and Polish versions underwent the required translation procedure pursuant to the EORTC Quality of Life Group [29]. The internal consistency of the scale was found to be high (Cronbach's alpha above 0.87 for each subscale) [30, 31]. The scale comprises 30 items (list of reasons why people engage in physical activities‚ sports, and exercise) in 5 categories: interest/enjoyment (referred to as enjoyment), competence, appearance, fitness, and social factors. The categories were assessed on a 7-point Likert scale (1 = “not at all true for me” to 7 = “very true for me”). In each category, the participants were segregated according to the median into low and high motivated individuals, separately for boys and girls.

The structure of weekly PA was determined by the Czech and Polish versions of the International Physical Activity Questionnaire-Long form (IPAQ-LF) [32, 33]. Both language versions underwent the required translation procedure and were in the long term empirically verified in previous studies [34, 35]. Pearson's correlation coefficient of concurrent validity between total PA (METs-min) and weekly step counts ranged from *r* = 0.231 to 0.283. Cronbach's alpha, as an indicator of internal consistency reliability, was 0.848 for the Polish version and 0.845 for the Czech version. The IPAQ-LF questionnaire included PA types (school, transport, housework, home, recreation, vigorous, moderate, and walking) and time spent sitting. Contrary to the guidelines for the IPAQ-LF, METs-min of vigorous PA (VPA) was assessed using a multiple of six instead of the recommended eight to avoid overestimation of time spent by PA and to not disrupt the proportional structure of weekly PA that is as objective as possible; the average daily sum of minutes of PA, transport, and sitting was set at a maximum of 960 min/day, and the maximum number of METs-min per week was set at 16,000 METs-min/week. A total of 191 respondents were excluded because of noncompliance with predetermined criteria.

The weekly PA recommendations were in accordance with the IPAQ-long questionnaire [36] determined in compliance with the generally acknowledged recommendations [37]. Meeting the stringent PA recommendations required the achievement of 60 minutes of MVPA on at least five days a week (in at least one of the PA types specified in the questionnaire) and at the same time 20 or more minutes of VPA on three or more days a week (5 × 60 min MVPA + 3 × 20 min VPA) [38]. This PA recommendation was selected because the greatest statistically significant correlations were observed between the types of motivation and VPA in adolescents (enjoyment *r*_*p*_ = 0.313, competence *r*_*p*_ = 0.347, appearance *r*_*p*_ = 0.247, fitness *r*_*p*_ = 0.206, social *r*_*p*_ = 0.168, and sum motivation *r*_*p*_ = 0.315).

### 2.3. Procedure

The introductory session concerning the completion of the questionnaires was held in a school computer lab. All participants were registered in the “International Database for Research and Educational Support” (Indares) (http://www.indares.com/). They were informed of the methods for maintaining data confidentiality and feedback concerning the average research results. First, the participants completed the IPAQ-LF questionnaire, followed by the MPAM-R scale. For reporting purposes, the ten-year monitoring period was divided into five two-year periods (2010–2011, 2012–2013, 2014–2015, 2016–2017, and 2018–2019) to document the trend in behavior changes.

### 2.4. Data Analysis

Statistical analyses were performed with the help of software Statistica, version 13 (StatSoft, Prague, Czech Republic), and SPSS, version 25 (IBM Corp., Armonk. NY). Basic descriptive statistics were applied to characterize the sample (mean, standard deviation, median, and interquartile range); one-way ANOVA was applied to assess gender differences in PA motives and types of PA; Kruskal-Wallis test was applied to identify the differences between low and high motive boys and girls, and nonparametric Spearman's correlation coefficient was used to identify the associations between types of PA and motivation types. Differences in the responses were assessed using the Mann–Whitney *U* test. To identify the differences in meeting the PA recommendations, cross-tabulation and percentage difference tests were conducted. The data distribution in the assessment of weekly PA and PA motives was presented using categorized scatter plots. Binary logistic regression with the standard entry method (where all independent variables are simultaneously entered into the equation at the same time) was used to assess the likelihood of achieving PA recommendations. The *η*_p_^2^ and *η*^2^ effect size coefficients were evaluated as follows: 0.01 ≤ *η*_*p*_^2^ (*η*^2^) < 0.06 indicated a small effect size, 0.06 ≤ *η*_*p*_^2^ (*η*^2^) < 0.14 indicated a medium effect size, and *η*_*p*_^2^ (*η*^2^) ≥ 0.14 indicated a large effect size. Statistical significance was set at *p* < 0.05.

### 2.5. Ethics

The study was conducted in accordance with the WMA Declaration of Helsinki, and the protocol was approved by the Ethics Committee of the Faculty of Physical Culture at Palacký University Olomouc for research projects (No. MSM6198959221 and No. 37/2013). School management, parents, and participants confirmed their agreement to participate in the research by providing written consent.

## 3. Results

### 3.1. Characteristics of Physical Activity Motives and Physical Activity Types among Boys and Girls over Five Two-Year Periods

**Figure 1:**
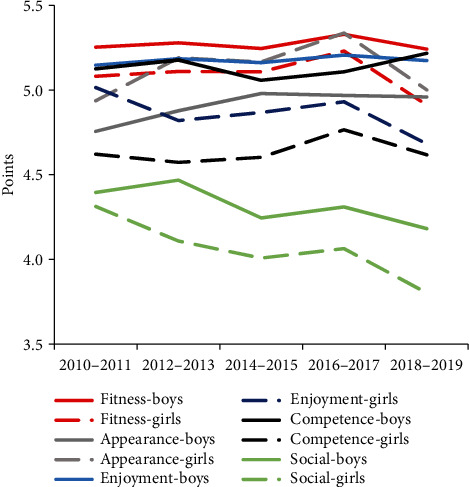
Gender-stratified estimated physical activity motives over five two-year periods.

During the time periods of this research, a decline was observed among girls in enjoyment motives (*F*_(4, 2513)_ = 3.66, *p* = 0.006, *η*_*p*_^2^ = 0.006), fitness motives (*F*_(4, 2513)_ = 3.08, *p* = 0.015, *η*_*p*_^2^ = 0.005), and social motives (*F*_(4, 2513)_ = 8.58, *p* < 0.001, *η*_*p*_^2^ = 0.013). However, an increase in appearance motives was observed (*F*_(4, 2513)_ = 5.41, *p* < 0.001, *η*_*p*_^2^ = 0.009) (Figure 1). No significant changes in PA motives were identified in boys during the study.

For the entire period, statistically significant gender differences were observed in enjoyment motives (*p* < 0.001), competence motives (*p* < 0.001), fitness motives (*p* < 0.001), and social motives (*p* < 0.001), among boys. Girls showed greater motivation than boys only in appearance motives (*p* < 0.001). Additionally, girls demonstrated greater motivation for PA than boys only in their responses to following motives: No. 17 to which they responded with the option, “Because I want to improve my appearance” (*U* = 6.15, *p* < 0.001, *η*^2^ = 0.024); No. 20, to which they responded, “Because I want to be attractive to others” (*U* = 1.88, *p* = 0.061, *η*^2^ = 0.002); No. 21, where they responded with “Because I want to meet new people” (*U* = 1.96, *p* = 0.049, *η*^2^ = 0.002); No. 24, where they responded with “Because I want to improve my body shape” (*U* = 8.46, *p* < 0.001, *η*^2^ = 0.046); and No. 27, with the option, “Because I will feel physically unattractive if I do not” (*U* = 2.10, *p* = 0.036, *η*^2^ = 0.003).

**Figure 2:**
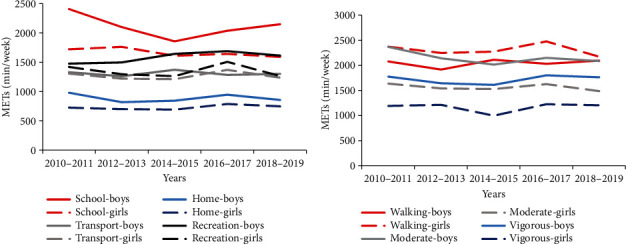
Structure of physical activity by implementation (a) and intensity (b) over five two-year periods.

Concerning the structure of PA, a decrease was observed only in recreation PA among girls over the two five-year periods (*F*_(4, 2513)_ = 2.41, *p* = 0.048, *η*_*p*_^2^ = 0.004) (Figure 2). In boys, no statistically significant changes in the types of PA were found during the study period. In total, a statistically significant gender difference was observed in favor of boys in school PA (*p* < 0.001), home PA (*p* < 0.001), recreation PA (*p* < 0.001), walking (*p* < 0.001), and moderate PA (*p* < 0.001), with the greatest difference being in VPA, where boys achieved 1,723 METs-min/week while girls reached 1,166 METs-min/week (*F*_(1, 4074)_ = 94.55, *p* < 0.001, *η*_*p*_^2^ = 0.023). It was found that only in the transport PA, the gender differences were not statistically significant (*p* = 0.741).

### 3.2. Differences in Weekly Physical Activity between Low and High Motivated Boys and Girls (by Types of Physical Activity and PA Motives)

The greatest statistically significant differences between low and high motivated individuals were found between recreation PA and all types of motivation in both sexes in the two countries (Table 2). It should be noted that only in recreational PA, low motivated Czech boys were found to be more active than low motivated Czech girls. This applies to all PA motives. The smallest impact of PA motives was observed in transport PA (except for appearance motivation in Polish boys).

The greatest impact on vigorous PA was observed in low and high motivated boys and girls in all types of motivation; however, statistically significant values were observed in all groups only in enjoyment, competence, and fitness motives (Table 3). Additionally, the differences between boys and girls were statistically significant in all low and high motivated groups in vigorous PA in competence motives. The smallest impact of PA motives was observed during walking. Girls reported more walking than boys but was statistically significant only for Polish low motivated girls compared with low motivated Polish boys in motives of enjoyment, competence, and appearance and compared with Czech low motivated girls in competence motives, as opposed to low motivated Czech boys.

### 3.3. Associations between Recreation PA and PA Motives and between Vigorous PA and PA Motives

**Figure 3:**
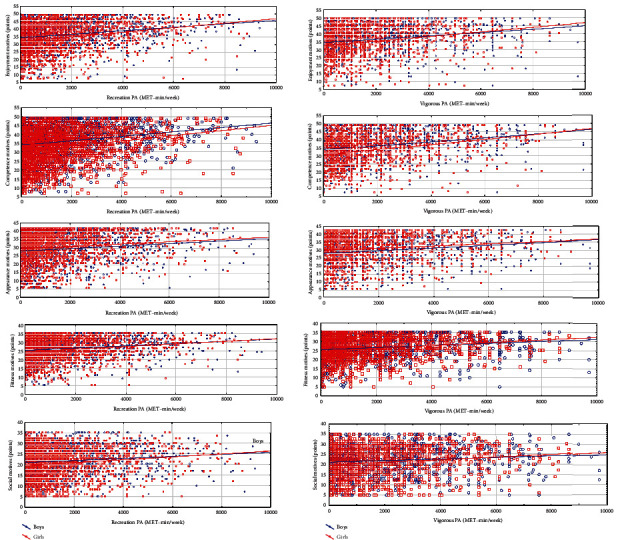
Correlations between recreation PA and PA motives (a) and between vigorous PA and PA motives (b) between boys and girls.

The greatest impact of PA motives on the types of PA was documented by the correlation analysis (Figure 3). In boys, the strongest associations were between recreation PA with enjoyment (*r*_*s*_ = 0.266) and competence motives (*r*_*s*_ = 0.263) and between VPA with enjoyment (*r*_*s*_ = 0.278) and competence motives (*r*_*s*_ = 0.313). In girls, the strongest associations were between recreation PA with enjoyment (*r*_*s*_ = 0.272) and competence motives (*r*_*s*_ = 0.253) and between VPA with enjoyment (*r*_*s*_ = 0.304) and competence motives (*r*_*s*_ = 0.313). The smallest correlations were reported for boys between social motives and recreation (*r*_*s*_ = 0.151) and vigorous PA (*r*_*s*_ = 0.128) and for girls and between appearance motives and recreation (*r*_*s*_ = 0.128) and vigorous PA (*r*_*s*_ = 0.139).

**Figure 4:**
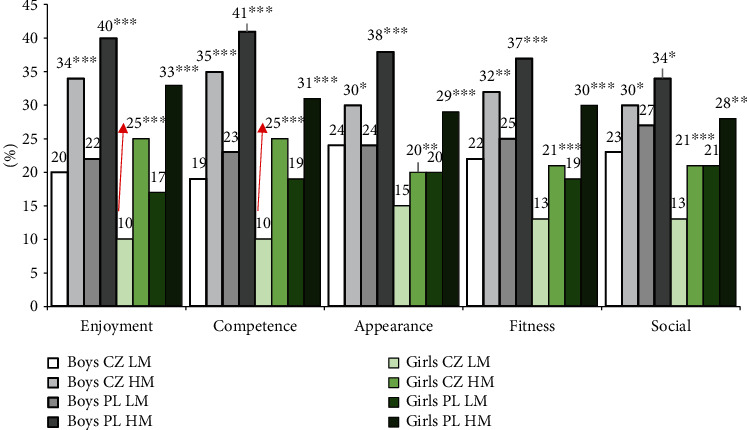
Achievement of PA recommendations (5 × 60 min moderate to vigorous PA + 3 × 20 min vigorous PA, no–yes) by low (LM: low motivation) and high (HM: high motivation) motivated Czech (CZ) and Polish (PL) boys and girls.

Greater motivation for PA had a positive effect on the achievement of PA recommendations in both low and high motivated boys and girls in all types of motivation (Figure 4). The greatest achievement of PA recommendation was observed in high motivated Polish boys, while the smallest achievement was found in low motivated Czech girls. The greatest impact on the achievement of PA recommendations was observed in all groups, caused by enjoyment and competence motives. Notable differences in the achievement of PA recommendations were observed between low and high motivated Czech girls in enjoyment (*χ*^2^ = 57.69, *p* < 0.001, *η*^2^ = 0.040) and competence motives (*χ*^2^ = 56.19, *p* < 0.001, *η*^2^ = 0.039), achieving 15 p.p., and in the achievement of PA recommendations by Polish high motivated girls in all PA motives (28% social motives to 33% enjoyment motives).

### 3.4. Predictors of Meeting PA Recommendations

Enjoyment, competence, and appearance motives in boys and girls increased the likelihood of achieving rigorous PA recommendations (VPA 3 × 20 min + MVPA 5 × 60 min) (Table 4). Adjusted moderator variables (country, age, BMI, and organized PA) involved in the model did not show a significant effect on the achievement of the PA recommendation neither in boys nor girls. Active participation in organized PA as the most significant moderator variable did not decrease the significance of the predictors of enjoyment, competence, and appearance motives for meeting the PA recommendations.

## 4. Discussion

### 4.1. Trends and Gender Differences in PA Motives and Types of PA

The results found that during the ten-year period, girls showed a decline in enjoyment, fitness, and social motives and an increase was observed only in appearance motives. A significant decrease in enjoyment motives was also observed by Abi Nader et al. [39] in both girls and boys. However, in the study, appearance motives were in the last position among other PA motives, while in Czech and Polish adolescents in the present study, the motives with the lowest assessment were social motives. This is consistent with the results of a previous Polish study [30].

Only in appearance motives were the girls more motivated to engage in PA than boys. In Polish girls, a greater appearance motive was observed by [40]. Generally, most studies confirm higher PA motivation among boys compared to girls, in both adolescents [41] and young adults [42]. However, a Norwegian study showed that girls had higher scores in intrinsic motives for sports participation, compared to boys, and that boys had higher scores in more extrinsic motives, but gender had no influence on motivation for sustained exercise [43].

The decrease in PA motives in girls is consistent with the decrease in recreation PA on average from 1,424 METs-min/week in the first period to 1,271 METs-min/week in the latest. Nevertheless, the values extracted in this study are greater than those reported in previous studies on Czech (1,146 METs-min/week) and Polish (990 METs-min/week) girls [44]. It should be noted that a statistically significant positive impact of all types of motivation was confirmed in recreation PA in both girls and boys. Naturally, it should be taken into account that these positive associations may also be affected by other factors. Aaltonen et al. [24] particularly highlighted the effect of genetic influence and an even greater influence of environmental factors. Similarly, Hankonen et al. [45] emphasized the effect of socioeconomic and other factors.

### 4.2. Differences in Weekly Physical Activity between Low and High Motivated Boys and Girls (by Types of PA and PA Motives)

The significant differences were observed between individuals with low and high motivation for PA, and the level of recreation PA in boys and girls emphasizes the importance of orientation of PA motives on preferred and pursued types of PA in leisure time. However, this requires respecting the preferences and possibilities for outdoor PA by less physically active adolescents in the context of their well-being [38] and provision of conditions/programs for less physically active adolescents and adolescents having a low socioeconomic status [46]. This also requires respecting the possibilities for active participation in organized PA by less physically active adolescents [34] and reversing the fact that four in every five adolescents do not experience the enjoyment motive and social, physical, and mental health benefits of regular PA [47].

The greatest impact on VPA was observed in low and high motivated boys and girls in all types of motivation; however, statistically significant values were observed in all groups only in enjoyment, competence, and fitness motives. The strong associations between enjoyment motives and VPA, as well as between competence motives and VPA in both genders, are a call for more intensive use of these types of motivation to support overall PA in adolescents. Similar conclusions were also formulated in a longitudinal study by Abi Nader et al. [39], who recommended that focus should be highlighted on enjoyment and competence motives in order to increase MVPA. According to Jakobsen and Evjen [43], intrinsic motives such as enjoyment and competence are vital for sustained exercise in Norwegian adolescent boys and girls. However, it also turns out that interventions to increase motivation for PA in schools may boost PA enjoyment motives, especially in girls [5]. Furthermore, perceived motor competence is an important factor to consider when attempting to promote an active and healthy lifestyle, primarily in students with low perceived motor competence, that is, with a lower level of motivation for PA [48]. It was confirmed that in 10- to 11-year-old Canadian children, targeting enjoyment and competence motives may be associated with increased participation in organized and group-based PA, as well as with an increased likelihood of meeting PA guidelines in youth [6].

The smallest impact of PA motives was observed in transport PA and walking (except for appearance motives in Polish boys). Simultaneously, transport to and from school represented as much as 36% of total daily MVPA on school days in high school [49], but interventions to increase the effects of walking to and from school are not sufficiently convincing [50]. It should be emphasized that active transport of Czech and Polish adolescents covers 22.5–24.9% of their overall weekly PA [51] and is the most efficient use of time for PA with respect to other school day segments [52]. Even the inclusion of a brief walking break during the routine school day helped direct the motivation to PA toward more intrinsic factors related to the possibility of staying with classmates and peer groups, as well as releasing surplus built-up energy [53]. The increase in autonomous motivation (i.e., intrinsic motivation, integrated, and identified regulation) in adolescents may improve active commuting to and from school [54].

Lawler et al. [55] drew attention to the dependence of psychological processes on the types of PAs performed. Girls actively participating in team or individual sports and boys participating in team sports demonstrated significantly higher self-determined motivational characteristics relative to other types of PA. This confirms the importance of respecting the differences in pursued but also preferred types of PA in boys and girls [38] in selecting the methods of motivating for PA or in selecting interventions to increase adolescents' PA and well-being.

### 4.3. Associations between Types of PA and Motivation in Boys and Girls and Achievement of PA Recommendations and Suggestion for Improvement

The observed achievement of PA by low and high motivated boys and girls emphasizes the importance of focusing on enjoyment and competence motives which are mostly associated with the achievement of PA recommendations. In order for students to achieve PA recommendations in school, they need to have intrinsic motivation supported by all stakeholders, including teachers, staff, and parents, to improve autonomy, relatedness, and competence for PA participation [56]. In the promotion of suitable motivation and types of PA in the school environment, it is necessary to respect the significant effect of the school's physical, social, and political environment on increasing PA and limiting sedentary behavior [57]. Rosenkranz et al. [58] added that it is also important to consider the characteristics of settings and leaders, along with insights from behavioral theory, setting theory, and evidence-based effective interventions. It is equally important to promote the experience of a positive affective response to acute PA to improve intrinsic motivation for PA [59].

For decision-making about the types of motivation for PA, it is also important to consider that wearables (such as smart wristbands or smart watches) increase motivation to be physically active via self-monitoring, goal setting, feedback, and competition. However, it is also important to note that children and youth often report technical problems and a new effect in using wearables, which may impact the long-term use of wearables [10]. Motivation for PA using technology allows greater individualization and respect for personal and individual characteristics, especially among students with lower levels of self-perception. The positive effects of interventions to promote PA using smartphone-based PA are found by Emberson et al. [60] but, at the same time, point out the limits in acting on intrinsic PA motivation.

### 4.4. Suggestions to Improve the Effectiveness of Motivation for PA in Girls and Low Motivated Adolescents

- The greatest emphasis should be placed on intrinsic motivation through enjoyment and competence motives for encouraging PAs in girls and low motivated adolescents
- Appearance motives should be used extensively to motivate girls for PA
- Regarding the negative effects of the pandemic, the postpandemic period should be used to improve adolescents' readiness for home-based PA, decreasing digital space time, and maintaining mental health and well-being
- In line with the findings of previous studies, the postpandemic era requires substantial changes in the approaches to PA motives and PA types
- Motivation for PA should be based on the knowledge of the preferences of PA types in the context of their achievement in the different segments of the school day, especially during leisure time
- The selection of motivation for PA should respect the specifics of the segments of the school day and weekdays

Future research should focus not only on the basic types of motivation and types of PA but also on the most preferred types of PA, especially among low motivated and less physically active adolescents. Future research should also focus on the characterization of the changes in the associations between PA motives and types of PA that occurred as a result of the negative impacts of the pandemic.

### 4.5. Strengths and Limitations

The strength of the study is the implementation of the ten-year research in clearly defined and identical settings in schools of both countries for the entire period of the research, which was enabled by the Indares web-based application.

The limit is the cross-sectional nature of the study, because it was not appropriate for practical and organizational reasons to carry out an annual random selection of participants in the school environment. In addition, it was impossible to carry out the research always on the same school day of the week, but instead, all school days of the week were considered almost evenly.

## 5. Conclusions

In the study, we sought to identify differences and associations between PA motives and types of PA in low and high motivated boys and girls and further to identify types of motives and PA that increase the likelihood of achieving PA recommendations among low motivated adolescents. The highest differences between both the low and high motivated boys and girls are apparent between recreation PA and all types of motivation. Enjoyment, competence, and appearance motives in both boys and girls increased the likelihood of achieving PA recommendations. The decrease in PA motives among girls calls for an increased attention to gender differences and the greater application of appearance motives in girls. Enjoyment, competence, and appearance motives in boys and girls increase the likelihood of achieving PA recommendations. Respecting and using the associations between PA motives and types of PA in low and high motivated boys and girls can support feelings of PA enjoyment, increase PA, support the achievement of PA recommendations, and positively affect adolescents' lifestyles. The role of the school environment for effective motivation for PA among low motivated and less physically active adolescents is irreplaceable in national, school, and local policies.

## Figures and Tables

**Table 1:** Sample characteristics.

Gender	Country	*n*	Age (years)		Weight (kg)		Height (cm)		PA (METs-min/day)		Sitting (min/day)	
			M	SD	M	SD	M	SD	M	SD	M	SD
Boys	Czech Republic	705	16.6	1.2	68.7	12.5	177.9	8.6	803	578	382	123
	Poland	853	16.3	0.7	67.7	12.7	176.8	7.6	879	604	367	151

Girls	Czech Republic	1444	16.8	1.2	59.1	9.2	167.1	6.7	679	508	395	118
	Poland	1074	16.3	0.7	56.9	8.6	165.9	6.1	773	556	371	143

*M*: mean; SD: standard deviation; PA: physical activity.

**Table 2:** Associations between the types of weekly PA (METs-min/week) and types of motivation in low and high motivated Czech and Polish boys and girls.

Types of motivation	Czech				Polish				*H*	*p*	*ŋ* ^2^
	Boys (*n* = 705)		Girls (*n* = 1444)		Boys (*n* = 853)		Girls (*n* = 1074)				
	Low Mot	High Mot	Low Mot	High Mot	Low Mot	High Mot	Low Mot	High Mot			
	Mdn (IQR)	Mdn (IQR)	Mdn (IQR)	Mdn (IQR)	Mdn (IQR)	Mdn (IQR)	Mdn (IQR)	Mdn (IQR)			
School PA											
Enjoyment	690 (2286)	1174 (3079)	549 (1864)	719 (2320)	1313 (3351)	1998 (4118)	956 (2672)	1601 (3454)	139.17^d,e^	<0.001	0.032^∗^
Competence	690 (2304)	1120 (2969)	540 (1835)	765 (2227)	1440 (3500)	1911 (4065)	952 (2723)	1560 (3544)	134.71^a,d,e^	<0.001	0.031^∗^
Appearance	690 (2555)	1079 (2624)	542 (1938)	715 (2303)	1321 (3355)	2160 (4100)	1188 (2873)	1187 (3227)	115.81^f^	<0.001	0.027^∗^
Fitness	707 (2455)	932 (2850)	563 (1935)	665 (2244)	1393 (3550)	1862 (3867)	973 (2812)	1511 (3450)	122.47^d,e^	<0.001	0.028^∗^
Social	658 (2268)	1155 (3095)	565 (1980)	650 (2071)	1515 (3758)	1715 (3776)	1054 (2599)	1438 (3379)	123.01^a,e^	<0.001	0.029^∗^

Transport PA											
Enjoyment	675 (1370)	757 (1378)	693 (1349)	825 (1539)	693 (1788)	990 (2126)	693 (1535)	774 (1782)	22.27	0.002	0.004
Competence	693 (1340)	756 (1451)	693 (1329)	792 (1396)	720 (1925)	930 (1956)	714 (1593)	743 (1832)	8.48	0.292	0.001
Appearance	693 (1320)	705 (1392)	693 (1320)	816 (1535)	611 (1646)	1071 (2061)	792 (1716)	693 (1755)	21.17^c^	0.004	0.003
Fitness	684 (1323)	746 (1332)	693 (1370)	809 (1389)	693 (1881)	930 (1928)	693 (1716)	774 (1698)	11.86	0.105	0.001
Social	693 (1311)	743 (1452)	693 (1320)	792 (1386)	743 (1749)	924 (2163)	707 (1485)	746 (1900)	4.78	0.687	0.001

Home PA											
Enjoyment	435 (1150)	545 (1494)	270 (740)	360 (785)	385 (985)	360 (1010)	365 (790)	420 (848)	30.86	<0.001	0.006
Competence	333 (1073)	630 (1513)	280 (750)	360 (776)	375 (988)	360 (1010)	325 (775)	480 (860)	46.88^a,d,f^	<0.001	0.010^∗^
Appearance	375 (1080)	613 (1443)	300 (750)	360 (790)	355 (1013)	390 (960)	375 (785)	420 (865)	27.18	<0.001	0.005
Fitness	368 (1093)	630 (1380)	270 (760)	374 (760)	360 (973)	378 (1045)	320 (818)	485 (820)	47.59^a^	<0.001	0.010^∗^
Social	424 (1170)	570 (1510)	280 (790)	360 (720)	355 (865)	380 (1165)	360 (810)	450 (800)	29.47	<0.001	0.006

Recreation PA											
Enjoyment	837 (1630)	1569 (2379)	657 (1232)	1392 (2034)	462 (1485)	1389 (2673)	429 (1230)	955 (2052)	298.62^a,b,c,d,e,^	<0.001	0.072^∗∗^
Competence	806 (1601)	1650 (2388)	682 (1292)	1326 (1978)	489 (1519)	1422 (2771)	462 (1179)	980 (2096)	276.08^a,b,c,d,e^	<0.001	0.066^∗∗^
Appearance	1002 (1958	1302 (2526)	788 (1535)	1098 (2772)	534 (1610)	1169 (2724)	537 (1284)	809 (1912)	130.10^a,b,c,d,e^	<0.001	0.030^∗^
Fitness	922 (1878)	1476 (2437)	742 (1526)	1155 (1880)	534 (1569)	1169 (2715)	500 (1252)	855 (1913)	155.39^a,b,c,d,e^	<0.001	0.036^∗^
Social	949 (1869)	1485 (2317)	756 (1453)	1179 (1941)	616 (1727)	931 (2620)	577 (1380)	803 (1946)	119.63^a,b,c,d,e^	<0.001	0.028^∗^

Note: *H*: Kruskal-Wallis ANOVA; *p*: significance; *ŋ*^2^: effect size coefficient; PA: physical activity; ^∗^0.01 ≤ *ŋ*^2^ < 0.06 small effect size; ^∗∗^0.06 ≤ *ŋ*^2^ < 0.14 medium effect size; ^∗∗∗^*ŋ*^2^ ≥ 0.14 large effect size; ^a^Czech boys low motivation–Czech boys high motivation; ^b^Czech girls low motivation–Czech girls high motivation; ^c^Polish boys low motivation–Polish boys high motivation; ^d^Polish girls low motivation–Polish girls high motivation; ^e^Czech boys low motivation–Czech girls low motivation; ^f^Czech boys high motivation–Czech girls high motivation; ^g^Polish boys low motivation–Polish girls low motivation; ^h^Polish boys high motivation–Polish girls high motivation.

**Table 3:** Associations between weekly PA of different intensities (METs-min/week) and types of motivation in low and high motivated Czech and Polish boys and girls.

Types of motivation	Czech				Polish				*H*	*p*	*ŋ* ^2^
	Boys (*n* =705)		Girls (*n* =1444)		Boys (*n* =853)		Girls (*n* =1074)				
	Low Mot	High Mot	Low Mot	High Mot	Low Mot	High Mot	Low Mot	High Mot			
	Mdn (IQR)	Mdn (IQR)	Mdn (IQR)	Mdn (IQR)	Mdn (IQR)	Mdn (IQR)	Mdn (IQR)	Mdn (IQR)			
Vigorous PA											
Enjoyment	720 (1620)	1530 (2430)	150 (900)	840 (2070)	630 (2160)	1800 (3150)	225 (1140)	1080 (2595)	409.82^a,b,c,d,g,h^	<0.001	0.099^∗∗^
Competence	615 (1650)	1560 (2190)	165 (900)	840 (2055)	720 (2310)	1800 (3360)	240 (1260)	1050 (2700)	406.42^a,b,c,d,g,h^	<0.001	0.098^∗∗^
Appearance	720 (2160)	1260 (2250)	360 (1380)	540 (1710)	840 (2350)	1620 (3120)	420 (1650)	720 (2250)	169.52^a,c,d,h^	<0.001	0.04^∗^
Fitness	720 (2130)	1440 (2340)	270 (1080)	720 (1890)	735 (2430)	1560 (3000)	360 (1440)	930 (2460)	247.28^a,b,c,d,g,h^	<0.001	0.052^∗^
Social	735 (2070)	1320 (2370)	270 (1080)	720 (2160)	1080 (2520)	1260 (3120)	420 (1620)	840 (2520)	197.39^a,b,d,g^	<0.001	0.047^∗^

Moderate PA											
Enjoyment	1110 (2090)	1740 (2759)	690 (1415)	978 (1828)	1440 (2840)	1680 (3020)	973 (1870)	1260 (2108)	162.48^a,b,g^	<0.001	0.038^∗^
Competence	1040 (2033)	1820 (2780)	690 (1480)	953 (1715)	1560 (2806)	1670 (3113)	930 (1845)	1320 (2160)	169.18^a,b,d,e,g^	<0.001	0.040^∗^
Appearance	1110 (2130)	1680 (2573)	740 (1450)	900 (1700)	1438 (2828)	1725 (3090)	1120 (1835)	1210 (2255)	137.38^a,h^	<0.001	0.032^∗^
Fitness	1060 (2220)	1680 (2580)	720 (1515)	925 (1610)	1411 (2808)	1770 (3100)	980 (1853)	1270 (2170)	159.31^a,d,g^	<0.001	0.037^∗^
Social	1260 (2013)	1590 (2710)	730 (1580)	893 (1625)	1405 (2840)	1785 (3080)	1013 (1870)	1260 (2120)	138.48^g^	<0.001	0.032^∗^

Walking											
Enjoyment	1287 (2360)	1477 (2533)	1617 (2475)	1658 (2673)	1139 (2739)	1568 (2723)	1403 (2574)	1832 (2879)	51.37^c,g^	<0.001	0.011^∗^
Competence	1279 (2269)	1518 (2525)	1667 (2657)	1584 (2549)	1155 (2789)	1551 (2706)	1485 (2607)	1832 (2805)	43.08^g^	<0.001	0.009
Appearance	1386 (2327)	1452 (2558)	1518 (2376)	1716 (2772)	1139 (2525)	1617 (3053)	1568 (2558)	1716 (2904)	51.16^c,g^	<0.001	0.011^∗^
Fitness	1353 (2384)	1452 (2525)	1625 (2508)	1634 (2640)	1188 (2764)	1403 (2706)	1502 (2558)	1815 (2855)	37.15	<0.001	0.007
Social	1320 (2211)	1518 (2706)	1617 (2574)	1650 (2607)	1254 (2574)	1436 (2970)	1485 (2426)	1782 (2921)	37.81	<0.001	0.008

Note: *H*: Kruskal-Wallis ANOVA; *p*: significance; *ŋ*^2^: effect size coefficient; PA: physical activity; ^∗^0.01 ≤ *ŋ*^2^ < 0.06 small effect size; ^∗∗^0.06 ≤ *ŋ*^2^ < 0.14 medium effect size; ^∗∗∗^*ŋ*^2^ ≥ 0.14 large effect size; ^a^Czech boys low motivation–Czech boys high motivation; ^b^Czech girls low motivation–Czech girls high motivation; ^c^Polish boys low motivation–Polish boys high motivation; ^d^Polish girls low motivation–Polish girls high motivation; ^e^Czech boys low motivation–Czech girls low motivation; ^f^Czech boys high motivation–Czech girls high motivation; ^g^Polish boys low motivation–Polish girls low motivation; ^h^Polish boys high motivation–Polish girls high motivation.

**Table 4:** Odds ratios for meeting the recommendation (3 × 20 min vigorous PA and 5 × 60 min moderate to vigorous PA) in boys and girls by low and high physical activity motives (enjoyment, competence, appearance, fitness, and social).

	Category	Model 1				Model 2			
		Boys		Girls		Boys		Girls	
		OR (95% CI)	*p*	OR (95% CI)	*p*	OR (95% CI)	*p*	OR (95% CI)	*p*
PA motives									
Enjoyment	Low								
	High	1.52 (1.13–2.05)	0.005^∗∗^	2.04 (1.56–2.67)	<0.001^∗∗∗^	1.43 (1.06–1.93)	0.018^∗^	1.99 (1.52–2.62)	<0.001^∗∗∗^
Competence	Low								
	High	1.71 (1.27–2.31)	<0.001^∗∗∗^	1.46 (1.12–1.91)	0.005^∗∗^	1.67 (1.23–2.56)	0.001^∗∗^	1.37 (1.04–1.79)	0.024^∗^
Appearance	Low								
	High	1.29 (1.00–1.66)	0.046^∗^	1.25 (1.01–1.55)	0.043^∗^	1.30 (1.01–1.67)	0.044^∗^	1.27 (1.02–1.57)	0.030^∗^
Fitness	Low								
	High	0.95 (0.71–1.27)	0.719	0.48 (0.75–1.24)	0.780	0.94 (0.70–1.26)	0.69	0.96 (0.74–1.24)	0.754
Social	Low								
	High	0.95 (0.74–1.22)	0.683	0.98 (0.78–1.22)	0.845	0.93 (0.72–1.19)	0.549	0.94 (0.75–1.18)	0.575

Moderator variables									
Country	Czech								
	Poland					0.81 (0.64–1.02)	0.068	1.50 (1.23–1.83)	0.001^∗∗^
Age	15-16								
	17–19					0.88 (0.70–1.11)	0.289	0.87 (0.71–1.07)	0.182
BMI	Normal								
	Overweight					0.91 (0.64–1.29)	0.600	0.67 (0.44–1.01)	0.053
Organized PA	No								
	Yes					1.75 (1.30–2.35)	<0.001^∗∗∗^	1.77 (1.37–2.27)	<0.001^∗∗∗^

OR: odds ratio; CI: confidence interval; ^∗^*p* < 0.05,  ^∗∗^*p* < 0.01,  and^∗∗∗^*p* < 0.001; PA: physical activity; Model 1: PA motives—enjoyment, competence, appearance, fitness, and social; Model 2: adjusted for country, age, BMI, and organized physical activity.

## Data Availability

The data that support the findings of this study are available from the corresponding author upon reasonable request.

## Abbreviations

PA: Physical activity
VPA: Vigorous physical activity
MVPA: Moderate to vigorous physical activity
BMI: Body mass index
MET: Metabolic equivalent
IPAQ-LF: International Physical Activity Questionnaire-Long form
MPAM-R: Motives for Physical Activity Measure-Revise
LM: Low motivation
HM: High motivation.
